# The influence of simulated rainfall on the removal of retained particulate matter on the surface of lawn grass leaves

**DOI:** 10.3389/fpls.2025.1622690

**Published:** 2025-07-29

**Authors:** Junrui Wang, Weihan Kong, Haimei Li, Yu Liu, Xiao Guo

**Affiliations:** College of Landscape Architecture and Forestry, Qingdao Agricultural University, Qingdao, Shandong, China

**Keywords:** simulated rainfall, turfgrass, particulate matter, wash-off rates, leaf surface structure

## Abstract

Rainfall can wash the surface atmospheric particulate matter (PM) into the soil, and restore the PM retention function of the turfgrass blades. The dynamic process of PM removal on turfgrass blades concerning rainfall intensity and duration was investigated, and the relationship between rainfall, leaf surface structure, and the rate of foliar PM removal was established. Seven turfgrass species (*Liriope* sp*icata*, *Lolium perenne*, *Festuca elata*, *Poa pratensis*, *Zoysia sinica*, *Cynodon dactylon* and *Agrostis stolonifera*) were examined in simulated rainfall experiments with total rainfall amounts of 16 mm, rainfall intensities of 10, 15, and 20 mm·h^–1^, and sampling intervals of 12, 8, and 6 min, respectively. The highest wash-off rates for foliar TSP, PM>10, PM2.5-10, and PM2.5 among the test plants were 84.05%, 87.99%, 78.62%, and 79.31%, respectively, with *Liriope* sp*icata* and *Zoysia sinica* exhibiting higher wash-off rates. Higher rainfall intensity led to greater wash-off rates, requiring less time to reach maximum wash-off rates. It is important to note that rainfall did not completely remove foliar PM, and PM retention after 20 mm· h^–1^ rainfall was lower than that after 10 mm· h^–1^ rainfall. Additionally, particulate wash-off rates decreased with the increase in groove width, leaf hair length, and leaf hair width in the leaf surface structure. The present study provides a scientific foundation for quantitative investigations into PM removal by garden plants and offers guidance for selecting urban greening plants.

## 1 Introduction

With rapid economic development and urbanization, atmospheric particulate matter (PM) pollution has emerged as a critical issue affecting public health (Cong et al., 2020; Roy et al., 2020). The PM_10_ particles (PM with particle sizes <10 μm) enter the respiratory system, while PM_2.5_(PM with particle sizes <2.5 μm)is associated with cerebrovascular and respiratory diseases (Hopke et al., 2019). Ultrafine particles have been found to impact meteorological processes and the water cycle (Kwon et al., 2020). Ecological research also indicates a strong correlation between average PM concentrations and COVID-19 morbidity and mortality (De Angelis et al., 2021). Therefore, controlling PM pollution in urban air is a paramount environmental protection concern today (Dang et al., 2022; Tiwari et al., 2024).

Leaves have a PM retention threshold beyond which they no longer absorb PM (Memoli et al., 2020). Rainfall plays a crucial role in washing PM deposited on plant leaves into the soil (Xu et al., 2019; Ysebaert et al., 2021), thereby restoring the PM filtration function of the leaf surface (Zhang et al., 2019). The efficiency of rainfall in removing PM from plant leaf surfaces is higher during the early stages of rainfall, with larger particles being removed more effectively than fine or ultrafine particles (Yan et al., 2019; Zhou et al., 2020). Variations in particle retention capacities among different plant species result in differing removal rates of leaf surface PM by rainfall (Zhou et al., 2021).

Plant foliage exhibits a critical atmospheric PM retention threshold, beyond which leaf surfaces cease to accumulate additional particles (Liu et al., 2013; Memoli et al., 2020). Rainfall-mediated scouring effects effectively transfer PM from foliar surfaces to soil substrates (Xu et al., 2019; Ysebaert et al., 2021), thereby restoring the particulate filtration capacity of leaves (Zhang et al., 2019). Optimization of precipitation-driven PM removal mechanisms for enhanced atmospheric particulate mitigation has emerged as a key research priority (Zhou et al., 2021). Consequently, while most investigations focus on quantifying rainfall characteristics, the stochastic nature of natural precipitation necessitates the predominant use of simulated rainfall methodologies, with rainfall intensity (mm h^-1^) serving as the principal quantitative parameter (Xu et al., 2019; Yan et al., 2019). Empirical studies demonstrate proportional increases in PM removal efficiency with elevated rainfall intensity and extended precipitation duration (Weerakkody et al., 2018; Cai et al., 2019; Luan et al., 2019). The initial precipitation phase exhibits particularly pronounced PM clearance, with removal efficacy following a size-dependent sequence: coarse particles > fine particles > ultrafine particles (Przybysz et al., 2014; Yan et al., 2019; Zhou et al., 2020). Interspecific variations in foliar PM retention capacity further result in species-specific differentials in rainfall-induced PM removal (Zhou et al., 2021). Notably, Rodríguez-Germade et al. (2014) documented that *Platanus hispanica* foliage achieves significant particulate removal through hydraulic scouring during precipitation events.

This study uniquely investigates the dynamic wash-off process of PM from plant leaves and their retention thresholds under systematically varied rainfall intensities and durations. By concurrently establishing quantitative relationships between these rainfall parameters, specific foliar morphological characteristics, and PM removal efficiency, it provides novel, comprehensive data on how precipitation events dynamically influence plant PM retention capacity. This integrated approach offers new insights for precisely evaluating urban vegetation’s PM removal efficacy and optimizing species selection to enhance airborne particulate mitigation.

## 2 Materials and methods

### 2.1 Selection of plants

**Figure 1:**
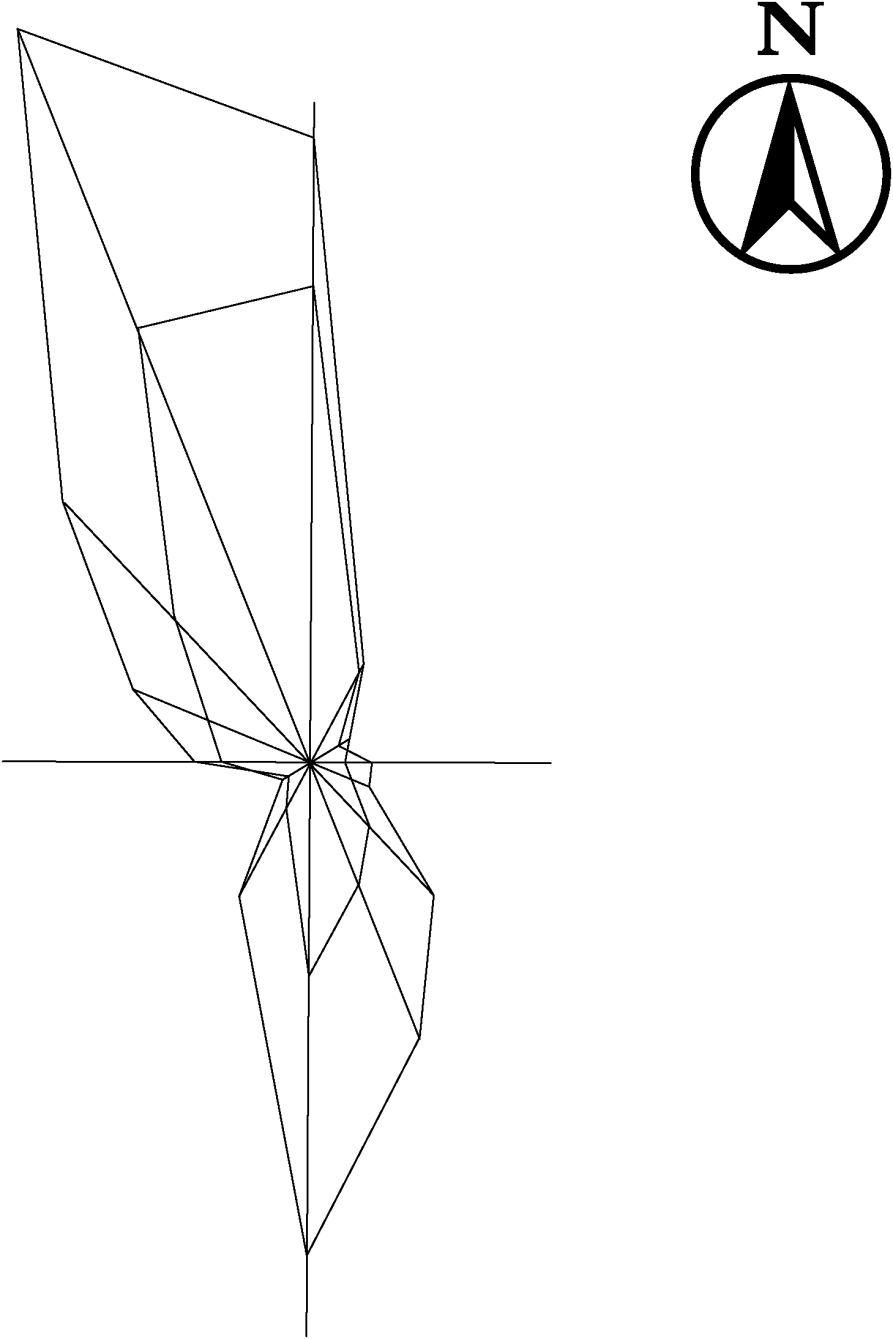
Wind direction map.

Seven common turfgrass species were selected found in Qingdao as the research subjects: *Liriope* sp*icata* (Thunb.) Lour, *Lolium perenne* L., *Festuca elata* Keng ex E. Alexeev, *Poa pratensis* L., *Zoysia sinica* Hance, *Cynodon dactylon* (L.) Pers., and *Agrostis stolonifera* L. In mid-July 2022, the seeds of *L. perenne*, *F. elata*, *P. pratensis*, *Z. sinica*, *C. dactylon*, and *A. stolonifera* were evenly sowed in pots (40 cm long, 20 cm wide, and 15 cm high). These pots contained a nutrient-rich soil mixture comprising grass charcoal, vermiculite, and Daejeon soil in a 1:1:1 volume ratio. Eight pots of each species were planted and allowed to grow into a lawn-like shape for preliminary study. *L.* sp*icata* was selected from the garden of Qingdao Agricultural University and transplanted into pots before the experiment. The plant material was pruned a week before the experiment to maintain the same height of about 15 cm. Prior to testing, the leaves were thoroughly washed and dried for one day. Subsequently, they were placed alongside the campus roadside of Qingdao Agricultural University (120°23’52” E, 36°19’5” N) for dust collection (Guo et al., 2023). The PM accumulation on leaf surfaces peaks after approximately 19 days, depending on the environment and plant species (Shao et al., 2019; Song et al., 2015). To prepare for the simulated rainfall experiment, the test plants were exposed to the roadside environment for 20 days without any rainfall events. The wind direction data during the experiment is shown in **Figure 1**.

### 2.2 Simulated rainfall

**Figure 2:**
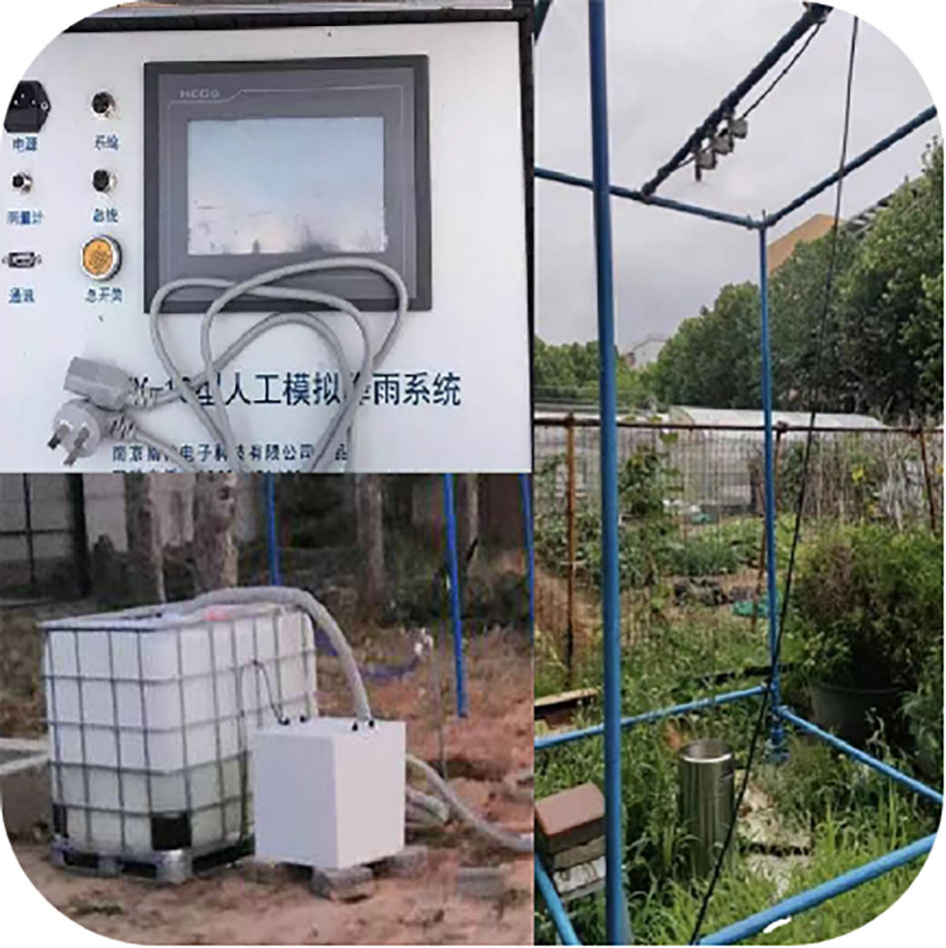
The original image of the rainfall simulation setting.

**Figure 3:**
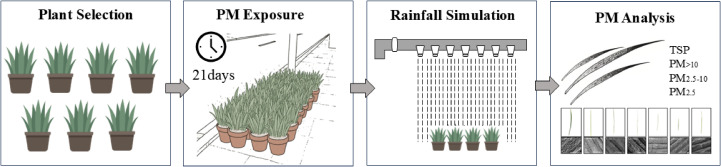
Schematic diagram of the simulated rainfall experiment.

The NLJY-10 artificial rainfall simulation system was used in the experiment (**Figure 2**). The rainfall height was 4 m and the simulated rainfall intensity was 6 to 240 mm·h^–1^. The rainfall characteristics closely resembled those of natural rainfall, with a rainfall uniformity exceeding 86%. Once the rainfall had stabilized, the test plants were placed with the highest simulated dust retention in the lower spray area of the simulated rainfall system. Three rainfall intensities were set at 10, 15, and 20 mm·h^–1^, respectively. According to the meteorological data of Qingdao, China, from 1987 to 2016, the average daily rainfall in Qingdao is about 2 mm in spring, summer and fall. Consequently, the sampling intervals were divided for the three rainfall intensities into 12, 8, and 6 min, respectively, to reflect the equivalent of 2 mm of rainfall at each interval. Under each rainfall intensity, with a total rainfall of 16 mm, 10 × 10 cm square leaves were collected at each sampling interval and placed them in labeled beakers. The filter membrane filtration method was used to determine the retained particles of various particle sizes on the leaf surface. The experimental flowchart is shown in **Figure 3**.

### 2.3 Measurement of foliar particles

Dust retention on leaf surfaces was expressed by quantifying the dust retained per unit leaf area, employing the filter membrane filtration quality method for assessment. Each experimental trial was conducted with six replicates. Distilled water was added to the beaker containing the leaf samples, submerging them for a duration of 2 h. Subsequently, the leaves underwent meticulous cleaning using a small brush and were then extracted with forceps, followed by rinsing of the leaf surfaces with a limited quantity of distilled water. The leaching solution was sequentially filtered through microporous filter membranes with diameters of 10 µm, 2.5 µm, and 0.22 µm. The filter membrane, housing trapped particles, was subsequently placed in an oven at 65°C until it reached a constant weight (with both measured values being less than 0.0002 g). This process allowed for the determination of particle mass within different size ranges on the leaf surface, including particle mass > 10 µm (designated as PM _> 10_), 10 ~ 2.5 µm (designated as PM_2.5–10_), and 0.22 ~ 2.5 µm (designated as PM_2.5_). The sum of these three values constituted the total suspended particulate matter (TSP). Following this, the dried leaves were carefully positioned on a scanner (CanoScan5600F, Canon Limited, Beijing, China) for scanning purposes, and the resulting digital images were imported into ImageJ for the calculation of leaf area, denoted as S (mm^2^). Subsequently, the dust retention of plant leaves per unit leaf area was determined as Q= (Q_2_ - Q_1_)·S^–1^.

Where: Q signifies the dust retention per unit leaf area (expressed in µg·mm^–2^), Q_2_ represents the mass of the filter membrane post-filtration (in µg), Q_1_ pertains to the mass of the filter membrane before filtration (in µg).

The wash-off amount of different particle sizes on the leaf surface by precipitation can be given as W_1_ = P-P_1_, and the wash-off rates can be given as W_2_ = (P - P_2_)·P^–1^.

Where: P represents the amount of dust retention per unit leaf area (µg· mm^–2^) before simulated rainfall, P_1_ is the amount of dust retention per unit leaf area (µg· mm^–2^) at the end of the sampling period, and P_2_ is the amount of dust retention per unit leaf area (µg· mm^–2^) after the sampling interval.

The retention threshold is defined as the minimum value of particle retention influenced by rainfall, and it is taken as the amount of dust per unit leaf area at the end of rainfall.

### 2.4 Determination of leaf surface structure

To determine the leaf surface structure, normal leaves were cut and immediately sealed in clean plastic bags to prevent damage to leaf hairs. Tissue blocks measuring 3 mm × 3 mm were taken from the middle of both sides of the vein using a new blade. These tissue blocks were placed into small glass bottles and fixed with FAA solution for over 4 h under vacuum. The samples underwent dehydration with a series of ethanol solutions (60%, 70%, 80%, 90%, and 100%), followed by replacement with tert-butanol for 10 min each time before freezing. The frozen plant samples were then put into a freeze-dryer for vacuum drying. Once completely dry, the samples were removed and coated with gold on the platform. The scanning electron microscope JSM-7500F (JEOL Ltd, Tokyo, Japan) was utilized to observe the surface structure of the plant leaves at various magnifications. Microstructural parameters of the leaf surface of the tested plants, such as groove ratio (groove projection area ratio), groove width, leaf hair density, leaf hair length, and leaf hair width, were observed and recorded. Subsequently, a correlation analysis was performed between these microstructure parameters and the particle wash-off rates of different particle sizes on the leaf surface of the tested plants under varying rainfall intensities.

### 2.5 Data analysis and processing

The data obtained from the experiment were processed using Excel 2016 software, analyzed using SPSS 25.0 (SPSS, IBM, USA) software, and visualized using Origin 2021 software. The elution rates of foliar particles with different particle sizes and species were compared by one-way analysis of variance (ANOVA) and two-way analysis of variance. Duncan’s method was used for multiple comparisons, the significance level of the difference was set at 0.05 (Guo et al., 2025).

## 3 Results

### 3.1 Cumulative dust retention before simulated rainfall

**Figure 4:**
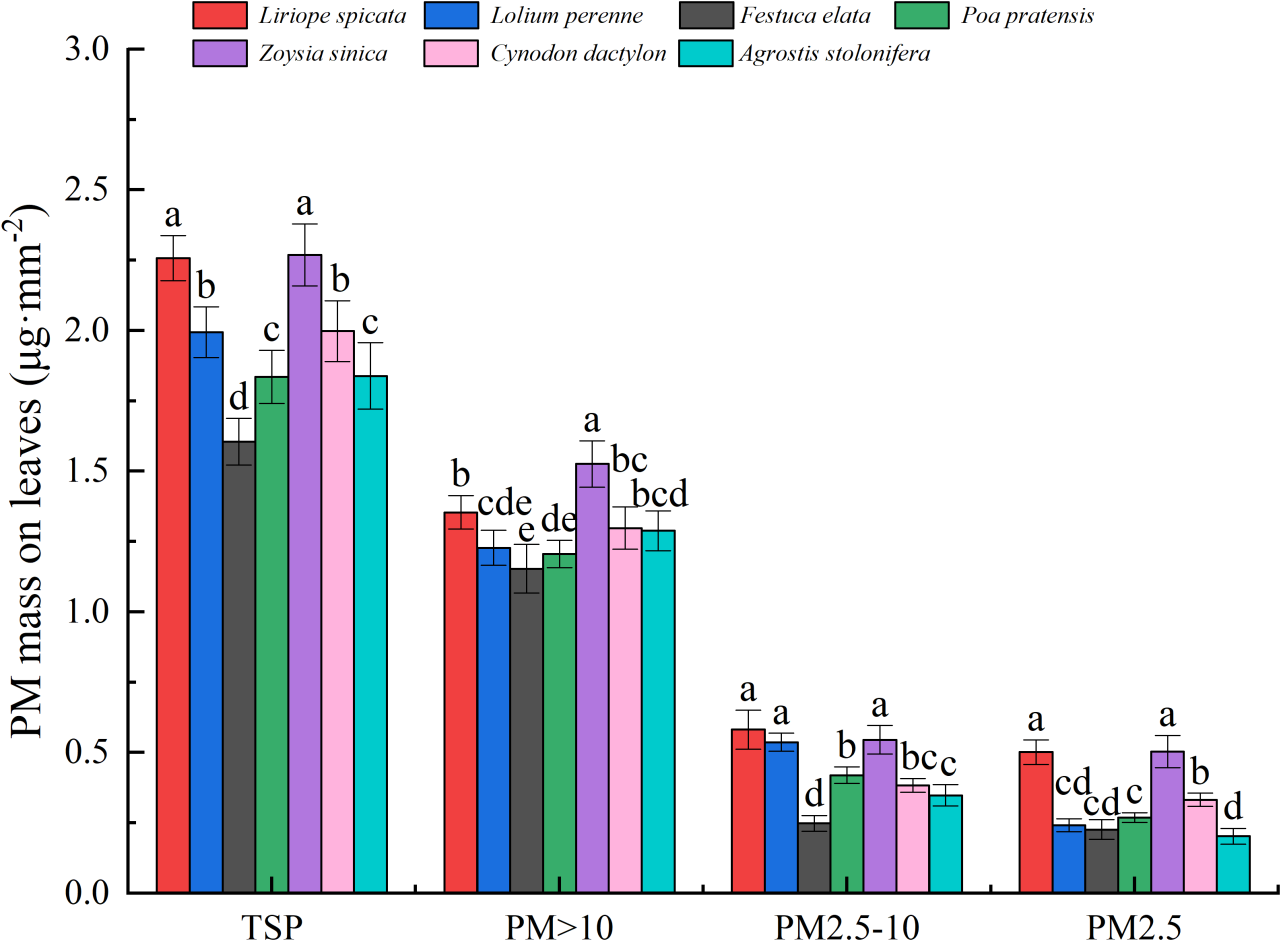
Cumulative retention of particles of varying sizes per unit leaf area in the tested plants. Values are means ± standard deviation (n = 6). Different lowercase letters indicate statistically significant differences in particulate retention among species (p < 0.05).

The PM_>10_ in foliar PM had the highest contribution to PM retention, followed by PM_2.5-10_, with PM_2.5_ contributing the least (**Figure 4**). Overall, the changes in TSP on the leaf surface ranged from 1.605 to 2.269 µg· mm^–2^, with *L.* sp*icata* and *Z. sinica* having the highest dust retention capacity. Leaf surface PM_>10_ for the tested plants varied from 1.153 to 1.526 µg· mm^–2^, with *Z. sinica* having the highest PM_>10_ retention capacity. Leaf surface retention of PM_2.5–10_ ranged from 0.248 to 0.581 µg· mm^–2^, with *L.* sp*icata*, *Z. sinica* and *L. perenne* being the highest. The range of leaf surface PM_2.5_ retention was 0.202 to 0.503 µg· mm^–2^, with *L.* sp*icata* and *Z. sinica* displaying the highest PM_2.5_ retention ability.

### 3.2 Effects of simulated rainfall on PM wash-off amount on the plant leaf surface

**Figure 5:**
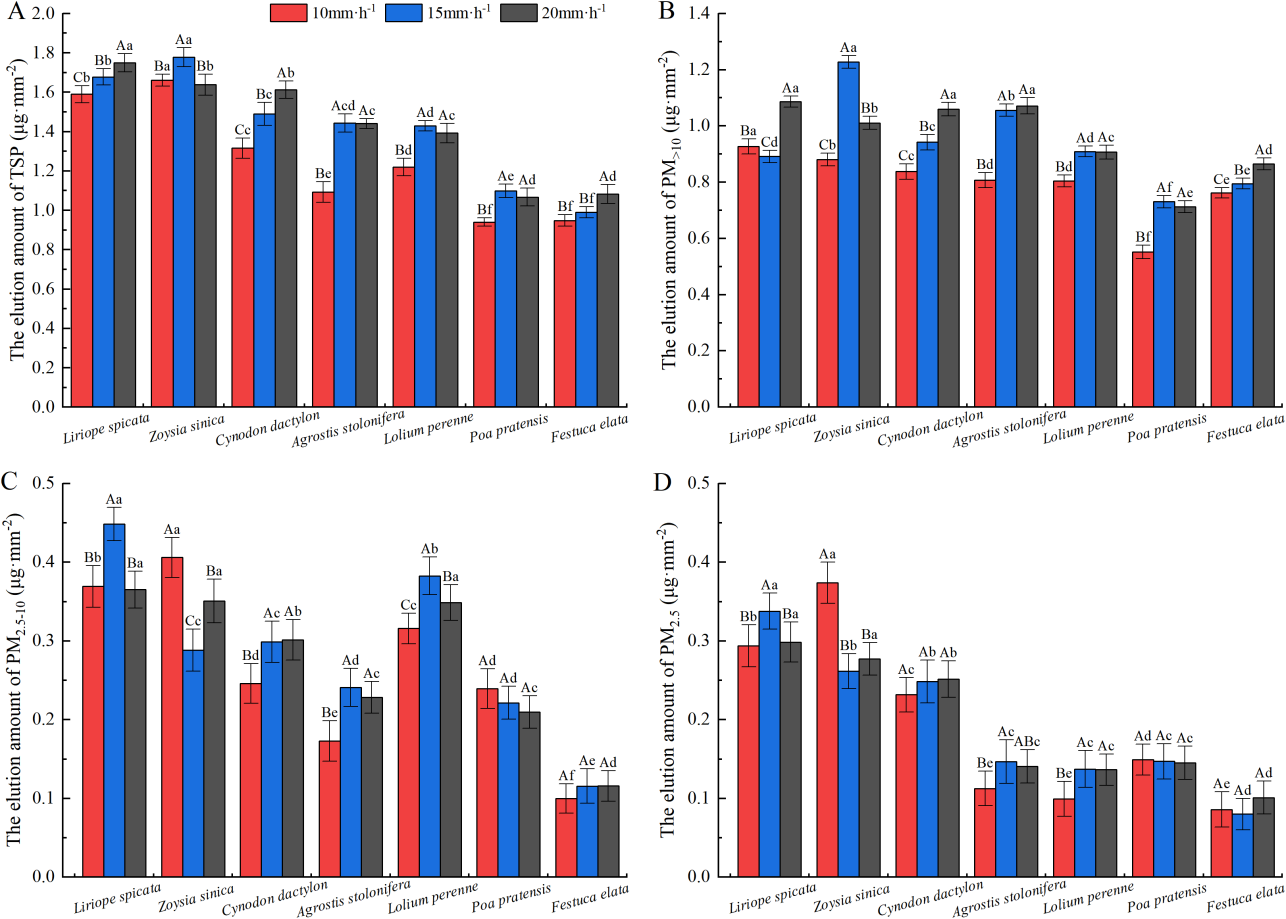
Particle elution of varying sizes on the leaf surfaces of the tested plants under varying rainfall intensities. Values are means ± standard deviation (n = 6). **(A)** The elution amount of TSP. **(B)** The elution amount of PM>10. **(C)** The elution amount of PM2.5-10. **(D)** The elution amount of PM2.5. Different lowercase letters indicate significant differences in particulate elution among species under the same rainfall intensity (p < 0.05), while different capital letters indicate significant differences in leaf particulate elution within the same species under different rainfall intensities (p < 0.05).

It was shown that higher rainfall intensity resulted in higher TSP and PM>10 wash-off amount for *L.* sp*icata*, *C. dactylon* and *F. elata*. (**Figure 5**). After 15 mm· h^–1^ and 20 mm· h^–1^ of rainfall, the wash-off amount of TSP and PM_>10_ for *A. stolonifera*, *L. perenne*, and *P. pratensis* were higher than those at 10 mm· h^–1^. There was no correlation between the amount of PM_2.5–10_ and PM_2.5_ on the leaf surface and rainfall intensity within the experimental range.

The foliar TSP, PM_>10_, PM_2.5–10_, and PM_2.5_ wash-off amount of the test plants ranged from 0.940 to 1.778 µg· mm^–2^, 0.551 to 1.228 µg· mm^–2^, 0.100 to 0.449 µg· mm^–2^, and 0.086 to 0.338 µg· mm^–2^, respectively. *Z. sinica* and *L.* sp*icata* exhibited high wash-off amount of foliar PM at all rainfall intensities. Additionally, *C. dactylon* and *A. stolonifera* had lower foliar PM_>10_ wash-off amount at 10 and 15 mm· h^–1^ rainfall intensities but higher PM_>10_ wash-off amount at 20 mm· h^–1^ rainfall intensity.

### 3.3 Effect of rainfall intensity on the wash-off rates of different particle sizes on foliage

**Figure 6:**
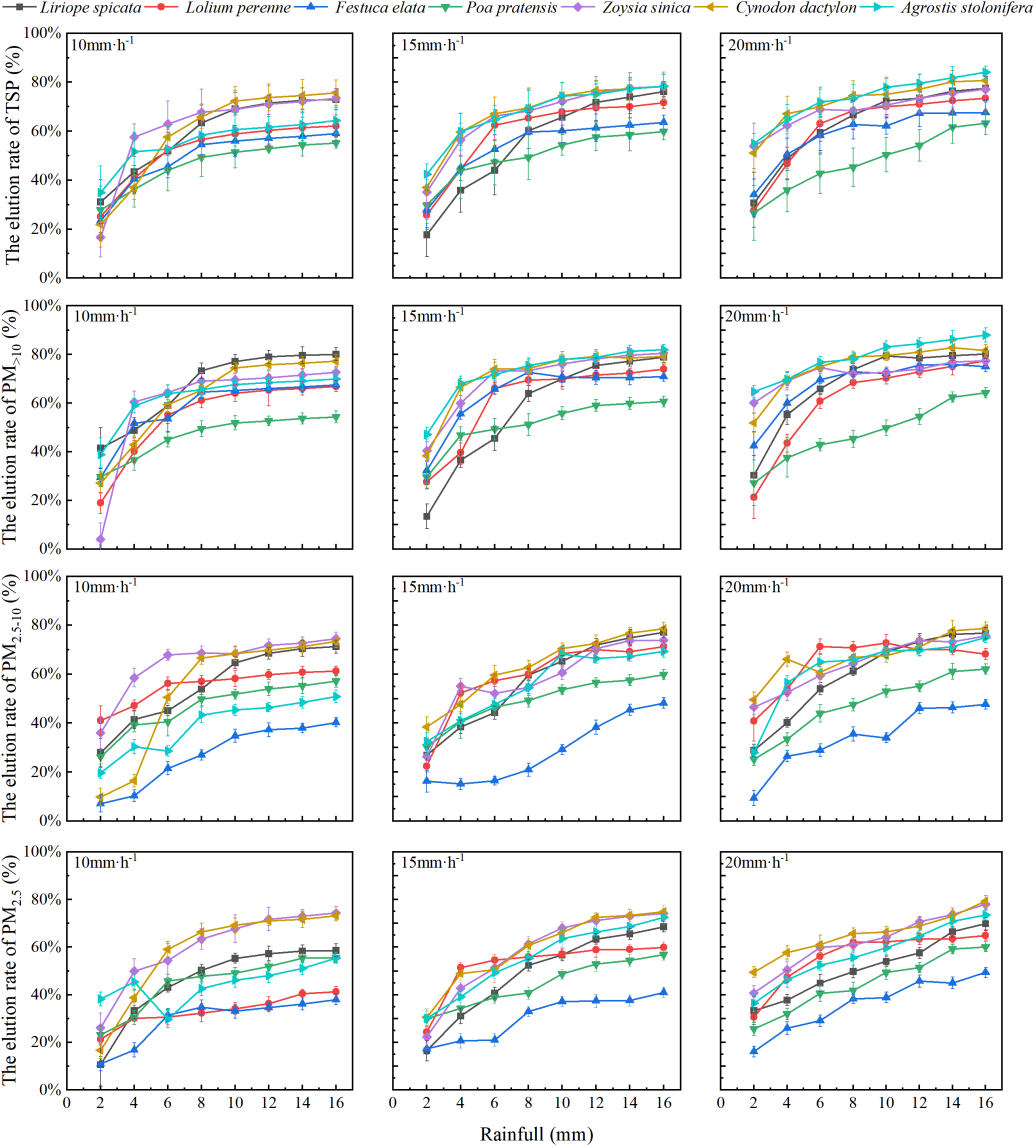
Variations in the elution rate of particulate matter with different particle sizes on the foliage of the test plants under various rainfall intensities. Values are means ±standard deviation (n = 6).

The wash-off rates of foliar PM from the test plants increased with increasing rainfall and exhibited two stages of growth: rapid and slow (**Figure 6**). At a rainfall intensity of 10 mm· h^–1^, the wash-off rates of PM of each particle size on the foliage of the test plants experienced rapid growth from 0 to 10 mm of rainfall, followed by a slower growth phase. At a rainfall intensity of 15 mm· h^–1^, the wash-off rates of each particle size on the foliage of the test plants showed rapid growth from 0 to 12 mm of rainfall, followed by a slower growth phase. At a rainfall intensity of 20 mm· h^–1^, the wash-off rates of each particle size on the foliage of the test plants remained in the rapid growth stage. The wash-off rates of PM from the foliage of the test plants after rainfall increased continuously to the maximum and also increased and then decreased before increasing to the maximum.

*L.* sp*icata*, *Z. sinica*, and *C. dactylon* exhibited high wash-off rates of PM of all particle sizes at all three rainfall intensities. The wash-off rates of *A. stolonifera* was low at a rainfall intensity of 10 mm· h^–1^, but the wash-off rates of all particle sizes on the foliage of *A. stolonifera* increased with higher rainfall intensity. The wash-off ranges of foliar TSP, PM _> 10_, PM_2.5–10_, and PM_2.5_ for the test plants during the experiment were 55.22% to 84.05%, 54.33% to 87.99%, 40.26% to 78.62%, and 38.00% to 79.31%, respectively. Furthermore, under different rainfall intensities, the wash-off rates of particles with different particle sizes on the leaf surface of the tested plants followed the order of PM_>10_ > PM_2.5–10_ > PM_2.5_, indicating that larger particles were easier to remove than smaller ones.

### 3.4 Effect of rainfall ephemeris on the wash-off rates of different particle sizes on foliage

**Figure 7:**
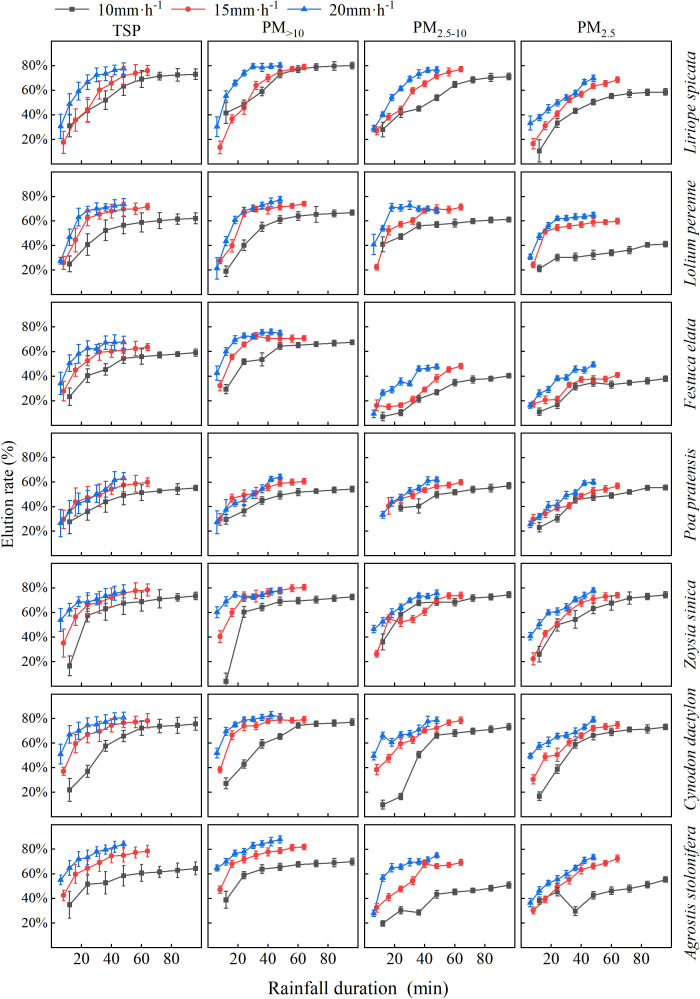
Alterations in the elution rates of particulate matter with different particle sizes on the foliage of the test plants in response to varying rainfall ephemeris at different rainfall intensities. Values are means ±standard deviation (n = 6).

With low rainfall intensities, the wash-off capacity of various particle sizes from the tested plants’ foliage was limited and lower compared to the wash-off rates during high-intensity rainfall events occurring simultaneously (**Figure 7**). Under the condition of 16 mm of rainfall, higher rainfall intensities resulted in greater wash-off rates of different particle sizes from the tested plants’ foliage, requiring shorter rainfall durations. Nevertheless, it was also observed that *L.* sp*icata* exhibited similar foliar particulate wash-off rates after prolonged low-intensity rainfall compared to short high-intensity rainfall.

### 3.5 Retention thresholds of foliar PM under different rainfall intensities

The foliar retention thresholds for PM at 10 mm· h^–1^ rainfall intensity were higher than those at 15 mm· h^–1^ and 20 mm· h^–1^ rainfall intensities, indicating that higher rainfall intensities led to more extensive wash-off amount of foliar PM (**Table 1**). The retention thresholds for TSP, PM_>10_, PM_2.5–10_, and PM_2.5_ in the test plants ranged from 0.273 to 0.763 µg· mm^–2^, 0.146 to 0.474 µg· mm^–2^, 0.076 to 0.201 µg· mm^–2^, and 0.051 to 0.207 µg· mm^–2^, respectively. *P. pratensis* had the highest TSP and PM _> 10_ retention thresholds, while *L. perenne* and *L.* sp*icata* had the highest PM_2.5–10_ and PM_2.5_ retention thresholds, respectively. The *A. stolonifera* had the lowest retention of PM of all particle sizes at a rainfall intensity of 20 mm· h^–1^.

**Table 1:** Retention thresholds of particulate matter with different particle sizes on the foliage of the test plants under varying rainfall intensities.

Species	Rainfall intensity (mm·h^–1^)	PM mass threshold on the blade (µg·mm^–2^)			
		TSP	PM_>10_	PM_2.5–10_	PM_2.5_
*Liriope* sp*icata*	10	0.587 ± .0.043cA	0.230 ± .0.027eB	0.149 ± .0.026bA	0.207 ± .0.027aA
	15	0.526 ± 0.041bcB	0.239 ± .0.021dB	0.133 ± .0.021abB	0.155 ± .0.023aB
	20	0.507 ± 0.047bB	0.267 ± .0.019bcA	0.111 ± .0.023bC	0.129 ± .0.026aC
*Lolium perenne*	10	0.742 ± .0.044aA	0.399 ± .0.021bA	0.201 ± .0.019aA	0.142 ± .0.022bA
	15	0.564 ± 0.025bB	0.318 ± .0.020bcB	0.154 ± .0.024aB	0.092 ± .0.023bcB
	20	0.501 ± .0.049bC	0.265 ± .0.024cC	0.162 ± .0.023aB	0.074 ± .0.020cdC
*Festuca elata*	10	0.656 ± .0.028bA	0.368 ± .0.018cA	0.148 ± .0.019bA	0.140 ± .0.022bA
	15	0.566 ± 0.028bB	0.326 ± .0.019bB	0.124 ± .0.022abcB	0.115 ± .0.020bB
	20	0.519 ± 0.048bC	0.288 ± 0.022bcC	0.127 ± 0.019bB	0.104 ± 0.021abC
*Poa pratensis*	10	0.763 ± .0.022aA	0.464 ± .0.024aA	0.179 ± .0.025abA	0.120 ± .0.020bA
	15	0.736 ± 0.034aA	0.474 ± .0.022aA	0.149 ± .0.021aB	0.113 ± .0.022bcA
	20	0.621 ± 0.046aB	0.396 ± 0.021aB	0.128 ± 0.021bC	0.096 ± 0.021bcB
*Zoysia sinica*	10	0.599 ± 0.030cA	0.331 ± .0.023dA	0.139 ± .0.025cA	0.129 ± .0.026bA
	15	0.491 ± 0.049cB	0.297 ± .0.023cB	0.102 ± .0.027cdB	0.091 ± .0.022bcB
	20	0.487 ± 0.054bB	0.296 ± 0.023bB	0.113 ± 0.028bB	0.078 ± 0.021bcdC
*Cynodon dactylon*	10	0.422 ± 0.052dA	0.247 ± 0.028eA	0.089 ± 0.025dA	0.085 ± 0.022cA
	15	0.413 ± 0.058dAB	0.248 ± 0.027dA	0.082 ± 0.026dA	0.083 ± 0.027cdA
	20	0.385 ± 0.045cB	0.238 ± 0.024dA	0.082 ± 0.026cA	0.066 ± 0.023dB
*Agrostis stolonifera*	10	0.602 ± 0.053cA	0.347 ± 0.027cdA	0.167 ± 0.026bA	0.088 ± 0.022cA
	15	0.395 ± 0.046dB	0.232 ± 0.022dB	0.107 ± 0.024bcdB	0.056 ± 0.028dB
	20	0.273 ± 0.025dC	0.146 ± 0.029eC	0.076 ± 0.020cC	0.051 ± 0.021dB

Values are means ± standard deviation (n = 6). Different lowercase letters indicate significant differences (*p* < 0. 05) in foliar particle retention of different species at the same precipitation intensity; different uppercase letters indicate significant differences (*p* < 0. 05) in foliar particle retention of the same species at different precipitation intensities.

### 3.6 The interdependence between precipitation volumes under varying rainfall intensities and the wash-off efficiency of PM across distinct particle-size fractions

As shown in **Figure 7**,the wash-off rates of PM across distinct size fractions exhibited exponential increases with cumulative precipitation, with t1 values under 20 mm h^-1^ rainfall intensity exceeding those observed at 10 mm h^-1^. Notably, the fitting equation for PM_2.5_ demonstrated the highest t1 value under the 20 mm h^-1^ intensity regime.

### 3.7 Relationship between wash-off rates of foliar PM and leaf surface structure under different rainfall intensities

**Figure 8:**
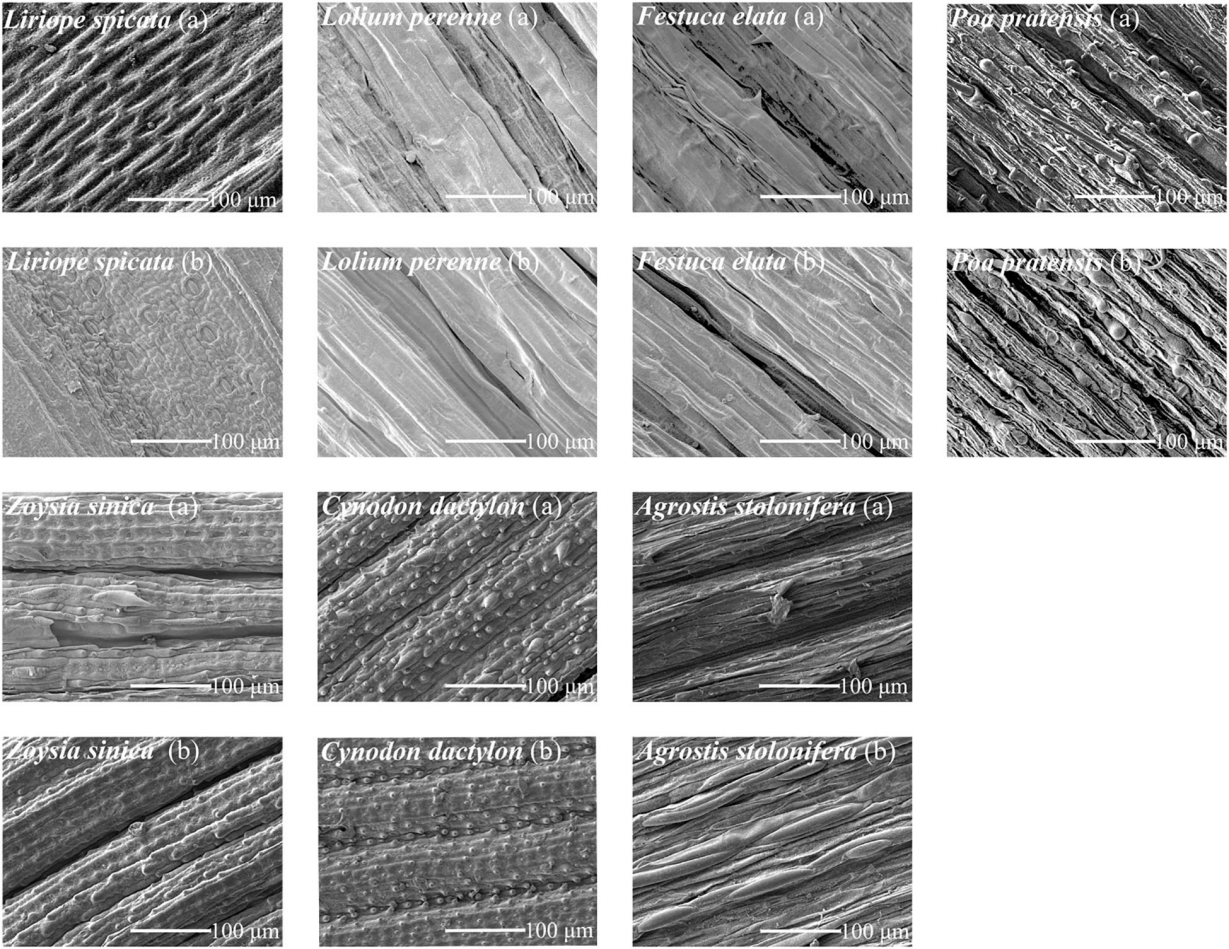
Scanning electron microscope images of the leaf surface structure of the test plants. Note: Images show the upper **(a)** and lower **(b)** leaf surfaces.

Observations and measurements of the surface microstructure of the leaf blades of seven turfgrass species were made using a scanning electron microscope (**Figure 8**, **Table 2**). *L.* sp*icata* leaf blades had no trichomes on the surface, a narrow grid of grooves, a waxy layer, and densely packed stomata. *L. perenne* leaf blades had no trichomes on the surface, wide grooves, and larger stomata. *F. elata* leaf blades had wide grooves with trichomes inside the grooves, and larger stomata. *P. pratensis* leaves have trichomes and bulbous projections, narrower grooves, and smaller stomata. *Z. sinica* leaves have multiple deeper grooves on both the adaxial and abaxial surfaces, with dense strips of projections between the grooves, and microscopic hairs between the grooves on the adaxial surface, resulting in smaller, denser stomata. *C. dactylon* leaves have multiple shallower grooves on both the adaxial and abaxial surfaces, with dense stripes between the grooves, and microhairs between the grooves on the adaxial surface, resulting in smaller, denser stomata. *A. stolonifera* leaf blades adaxially and abaxially have multiple shallow grooves, with dense globular projections between the grooves, also with microhairs between the adaxial grooves, smaller stomata, and lower stomatal density.

**Table 2:** Microstructural parameters of the blade surface.

Species	Groove Proportion (%)	Groove Width (µm)	Trichome Density (N·mm^–2^)	Trichome Length (mm)	Trichome Width (mm)
*Liriope* sp*icata*	0.57 ± 0.06a	27.62 ± 4.56c	–	–	–
*Lolium perenne*	0.34 ± 0.04c	72.31 ± 1.84a	–	–	–
*Festuca elata*	0.47 ± 0.03b	76.68 ± 6.05a	44.61 ± 8.92a	45.00 ± 4.06b	23.43 ± 0.78a
*Poa pratensis*	0.40 ± 0.04bc	50.32 ± 9.37b	47.58 ± 5.15a	119.47 ± 35.25a	22.44 ± 2.80a
*Zoysia sinica*	0.56 ± 0.09a	48.60 ± 2.13b	35.52 ± 8.88a	58.489.56 ± b	16.62 ± 1.46b
*Cynodon dactylon*	0.39 ± 0.03bc	23.95 ± 0.21c	56.23 ± 22.35a	38.60 ± 2.62b	17.07 ± 0.52b
*Agrostis stolonifera*	0.45 ± 0.03b	42.61 ± 5.12b	44.40 ± 8.88a	39.97 ± 13.89b	12.34 ± 1.00c

Leaf surface microstructural parameters are expressed as mean ± standard deviation, n = 6.

**Figure 9:**
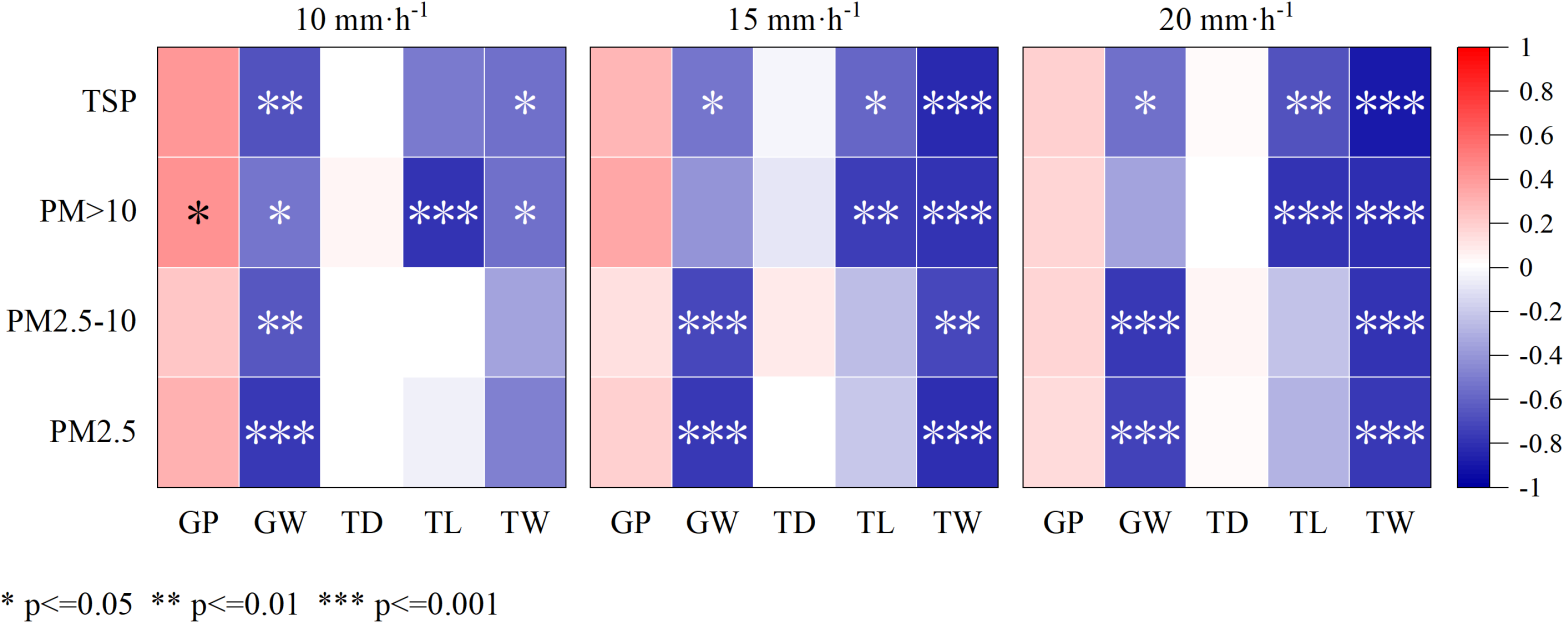
Correlation between the elution rate of foliar particulate matter and leaf surface structure under varying rainfall intensities. GP, Groove proportion; GW, Groove width; TD, Trichome density; TL, Trichome length; TW, Trichome width; and -, No data.

The larger the groove ratio, the greater the wash-off rates of PM _> 10_ at 10 mm· h^–1^ rainfall intensity (**Figure 9**). The wider the width of the grooves, the smaller the wash-off rates of TSP, PM_2.5-10_, and PM_2.5_ under the three rainfall intensities. The longer the leaf trichome length, the lower the wash-off rates of PM _> 10_. At rainfall intensities of 15 and 20 mm· h^–1^, the wider the width of the trichome, the lower the wash-off rates of PM.

With the increase of rainfall intensity, the correlation among TD pollutants did not change significantly. The difference is that the correlation between TSP and TL gradually shows significant differences with the increase of rainfall intensity. The correlation between TW and pollutants also shows a similar trend. The correlation between pollutants and GP and GW mostly changes from positive correlation to negative correlation, and the correlation and significance increase.

The differences in the correlations between different pollutants and the characteristics of each leaf may reflect the different influence mechanisms of rainfall intensity changes on the interaction between plant leaf characteristics and pollutants.

## 4 Discussion

### 4.1 Effect of rainfall on foliar particles

The capacity for foliar particulate removal is driven not only by rainfall but also by the duration of particulate accumulation on plant leaf surfaces, and the inherent maximum dust retention capacity of the plant leaves (Dzierzanowski et al., 2011; Gajbhiye et al., 2024). Species with rougher leaf surfaces tend to trap more PM (Sæbø et al., 2012; Tiwari et al., 2025), enabling them to wash away a higher quantity with the same amount of rainfall (Xu et al., 2017). In this study, *L.* sp*icata* and *Z. sinica* retained the most PM on their leaf surfaces under identical environmental conditions, leading to higher wash-off rates compared to other tested plants.

Rainfall exerts different effects on the wash-off rates of foliar PM from various plant types. For instance, It was found that 20 mm of rainfall removed 30% to 41% of the PM from red pine (Przybysz et al., 2014). It has also been found that cumulative rainfall amounts of 10.4 mm and 31.9 mm resulted in the removal of 28% and 48% of PM from the leaves of *Ligustrum lucidum* (Wang et al., 2015). Others demonstrated that the impact of rainfall on the removal of PM from the leaf surface was most pronounced for herbaceous plants, followed by shrubs, while trees exhibited the lowest efficiency in PM removal (Zhou et al., 2021). In this study, the removal of PM from turfgrass leaf surfaces during a total rainfall of 16 mm and a maximum rainfall intensity of 20 mm·h^–1^ ranged from 55.22% to 84.05%. This was notably higher than the wash-off rates observed for trees and shrubs under the same conditions (Xu et al., 2017).

### 4.2 Effect of rainfall intensity on foliar PM

It was found that the higher the rainfall intensity, the higher the wash-off rates of PM from the leaf surface for a range of rainfall intensities (Zhang et al., 2019; Zhou et al., 2021). This suggests that an increase in rainfall kinetic energy and raindrop diameter removes more leaf surface PM (Chen et al., 2017; Xu et al., 2017).

The highest wash-off rates was observed for PM_>10_, followed by PM_2.5–10_, while PM_2.5_ exhibited the lowest wash-off rates among the foliar PM of the test plants exposed to varying rainfall intensities. Numerous studies have reported similar trends (Wang et al., 2015; Zhou et al., 2020). This observation may be attributed to the distinct behaviors of different particle sizes on plant leaf surfaces. Coarse particles typically adhere in a manner that promotes retention or stagnation on the leaf surface, resulting in limited contact and easy removal by rainfall. In contrast, fine particles and ultrafine particles primarily adhere closely to the leaf surface, creating a larger contact area and thus resisting removal (Zhang et al., 2018). Additionally, variations in physicochemical properties among particle sizes have been reported, with some studies indicating that particles can form compounds that are resistant to removal by stormwater (Sæbø et al., 2012; Zhou et al., 2020).

Furthermore, a decrease in the wash-off rates of foliar PM from the test plants was observed following rainfall. This reduction can be attributed to the interplay between the decomposition of large particles and the aggregation of fine particles. During rainfall, larger particles may disintegrate into fine particles due to attraction and chemical reactions. Simultaneously, smaller particles may aggregate in the air and settle on the leaf surface. Consequently, the wash-off rates of foliar PM decreases during rainfall but subsequently increases as the rainfall persists (Cai et al., 2019; Hofman et al., 2016; Zhou et al., 2022).

### 4.3 Effect of rainfall ephemeris on the retention of foliar PM

The study has revealed that higher rainfall intensity is associated with increased wash-off rates and reduced rainfall duration requirements for various particle sizes on the leaf surface, assuming constant total rainfall. This observation aligns with previous research findings (Xu et al., 2017; Zhang et al., 2019). However, certain plant species with foliar particles exhibit similar wash-off rates under prolonged low-intensity rainfall and short high-intensity rainfall conditions. This phenomenon may be linked to the microstructure of the leaf surface (Zheng et al., 2005; Tiwari et al., 2023). Rough blade surfaces with greater wettability increase the contact time between rain and the blade surface under high-intensity rainfall, facilitating the removal of blade surface particles (Bussonnière et al., 2017). Consequently, for the same total rainfall, plants with rougher leaf surfaces can achieve higher wash-off rates of PM during short bursts of high-intensity rainfall (Zhang et al., 2019). In contrast, smooth blade surfaces allow low-intensity raindrops to rapidly pass through their water-repellent surfaces, efficiently carrying away particles (Neinhuis and Barthlott, 1998). Consequently, high particle wash-off rates can also be attained with extended periods of low-intensity rainfall (Zhang et al., 2019). In this investigation, the leaf surface of *L.* sp*icata* lacked leaf hairs and exhibited only small grid-like grooves, resulting in a relatively smooth surface. Consequently, PM_>10_ wash-off rates on the leaf surface of *L.* sp*icata* under low-intensity impact conditions closely resembled those achieved under high-intensity rainfall conditions.

### 4.4 Effect of leaf surface microstructure on the wash-off rates of PM

The microstructure of leaf blades significantly influences the wash-off rates of particles of varying sizes on the leaf surface. Before rainfall, leaf surface grooves retain a substantial amount of PM (Cai et al., 2017; Wang et al., 2015). Subsequent to rainfall onset, raindrops initially strike the blade, dislodging dust particles. Raindrops also pool in the foliar grooves to form runoff, which in turn flushes out PM trapped in the grooves. In this study, grooves affected the wash-off rates of PM>10 mainly at low rainfall intensities. Trench width exerts a reducing influence on the wash-off rates of PM_>10_ as rainfall intensity increases, while its impact on the wash-off rates of PM_2.5–10_ and PM_2.5_ intensifies.

In this study, the TSP retention threshold on the foliage of *P. pratensis*, which possesses trichomes, exceeded that of *L. perenne*, a grass lacking trichomes on its foliage. This finding aligns with previous research (Chen et al., 2021; Perini et al., 2017). While *Z. sinica*, *C. dactylon*, and *A. stolonifera*, all grasses with leaf surface trichomes, exhibited lower foliar TSP retention thresholds compared to *L. perenne*. Longer and more abundant leaf hairs on plants were found to enhance the adsorption and capture of PM (Lu et al., 2018). The *P. pratensis* offered the longest leaf hairs among the test plants, resulting in the highest retention threshold. The impact of leaf hair length on the TSP wash-off rates became increasingly significant with rising rainfall intensity, while the effect of leaf hair width on the wash-off rates of different particle sizes on the leaf surface strengthened with increasing rainfall intensity.

### 4.5 Limitations and prospects

However, this study did not measure the pre-existing PM retention on leaves prior to PM deposition experiments, nor did it analyze stomatal size. In future research, we will collect baseline leaf data before PM deposition sampling to enhance the reliability of experimental results. Additionally, we will conduct in-depth investigations into stomatal characteristics to explore their correlation with dust retention.

## 5 Conclusion

The flushing effect of the natural rainfall process on the PM retained on the plant foliage is the key to the restoration of the dust retention function of the plant, and both the rainfall characteristics and the plant species have an important influence on the flushing effect. Experiments show that the wash-off rates of PM gradually rises with increasing rainfall intensity. Higher rainfall intensity results in greater final wash-off rates of PM and shorter durations required to reach the corresponding wash-off rates. The highest wash-off rates for foliar TSP, PM_>10_, PM_2.5–10_, and PM_2.5_ among the test plants were 84.05%, 87.99%, 78.62%, and 79.31%, respectively, with *Liriope* sp*icata* and *Zoysia sinica* exhibiting higher wash-off rates. During rainfall, the particulate wash-off rates is reduced by the breakdown of large particles into fine particles. Compared with other plants, *Poa pratensis* has a stronger ability to retain PM. The groove width, leaf hair length, and leaf hair width of a plant have an inverse relationship with its PM wash-off rates.

This experiment studied the relationship between rainfall and the retention of PM on the surface of plant leaves. In practical applications, a scientific and reasonable layout of urban greening can be carried out based on the dust retention characteristics of plants and rainfall conditions. In areas with abundant rainfall, it is advisable to appropriately increase the area of green Spaces and the density of vegetation. By taking advantage of the scouring effect of rainfall and the dust-retaining function of plants, the air can be purified more effectively. Meanwhile, based on the elution law of PM on the surface of plant leaves caused by rainfall and the distribution characteristics of PM of different particle sizes, corresponding pollution control measures can be taken.

In future research, we will continue to study the changes in the dust retention capacity of plants under different seasons, climatic conditions and air quality conditions. At the same time, further explore in depth the differences in the absorption and purification capabilities of various plants for common harmful gases such as sulfur dioxide, nitrogen oxides, ozone, and volatile organic compounds, as well as the relationship between absorption efficiency and factors such as plant varieties, growth stages, and environmental conditions, so as to select targeted plants based on the dominant pollutant types in different cities.

## Data Availability

The raw data supporting the conclusions of this article will be made available by the authors, without undue reservation.
